# Flavonoids Enhance Lipofection Efficiency and Ameliorate Cytotoxicity in Colon26 and HepG2 Cells via Oxidative Stress Regulation

**DOI:** 10.3390/pharmaceutics14061203

**Published:** 2022-06-05

**Authors:** Die Hu, Shintaro Fumoto, Hirotaka Miyamoto, Masakazu Tanaka, Koyo Nishida

**Affiliations:** Graduate School of Biomedical Sciences, Nagasaki University, 1-7-1 Sakamoto, Nagasaki 852-8501, Japan; bb55720002@ms.nagasaki-u.ac.jp (D.H.); hmiyamoto@nagasaki-u.ac.jp (H.M.); matanaka@nagasaki-u.ac.jp (M.T.); koyo-n@nagasaki-u.ac.jp (K.N.)

**Keywords:** lipoplexes, antioxidants, reactive oxygen species, transfection, cell viability

## Abstract

The generation of reactive oxygen species (ROS) can affect cationic liposome-mediated transfection. In this study, we focused on a specific class of antioxidants, flavonoids, to investigate the transfection efficiency using cationic liposome/plasmid DNA complexes (lipoplexes) in 2D and 3D cultures of Colon26 and HepG2 cells, respectively. All tested flavonoids enhanced the transfection efficiency in 2D Colon26 and HepG2 cells. Among the tested flavonoids, 25 µM quercetin showed the highest promotion effect of 8.4- and 7.6-folds in 2D Colon26 and HepG2 cells, respectively. Transfection was also performed in 3D cultures of Colon26 and HepG2 cells using lipoplexes with quercetin. Quercetin (12.5 µM) showed the highest transfection efficiency at all transfection timings in 3D Colon26 and HepG2 cells with increased cell viability. Flow cytometry revealed that quercetin treatment reduced the population of gene expression-negative cells with high ROS levels and increased the number of gene expression-positive cells with low ROS levels in HepG2 cells. Information from this study can be valuable to develop strategies to promote transfection efficiency and attenuate cytotoxicity using lipoplexes.

## 1. Introduction

Gene therapy is an option for cancer treatment. The development of a safe and effective gene delivery system is important for successful gene therapy. Compared to viral vectors, non-viral vectors possess lower immunogenicity, indicating their potential for widespread application worldwide [1]. Among non-viral vectors, cationic liposome-based lipoplexes are the most commonly used because of their ease of preparation [2]. However, there are still drawbacks to the use of lipoplexes, such as insufficient transfection efficiency and cytotoxicity. The generation of high levels of reactive oxygen species (ROS) during transfection is one of the reasons for cytotoxicity [3]. In our previous study, edaravone, an efficacious antioxidant that scavenges ROS, enhanced the lipoplex gene expression levels in both HepG2 cells and mice [4]. The oil/water partition coefficient (log P) of drugs is important for cellular uptake and intracellular disposition. Compounds with moderate log P between –0.4 and 5.6, showed good permeation and absorption [5]. The log P value of edaravone (1.12 [4]) was moderate; thus, edaravone had a superior promotion effect on water-soluble antioxidant vitamins C and *N*-acetyl cysteine, and water-insoluble antioxidants vitamins A and E. Here, flavonoids, ubiquitous in food and plant sources, play an important role as antioxidants, gaining more advantages in terms of economic benefits and sustainable use. Flavonoids decrease ROS levels caused by H_2_O_2_ [6,7,8]. In addition, flavonoids also show a cell-protective effect, which leads to increased cell viability at certain concentrations [9,10]. To date, hepatoma and hepatic metastatic diseases from colorectal cancers are significant clinical problems [11,12]. Nanoparticulate drug delivery systems have been developed as promising therapeutic approaches [13,14]. We hypothesized that flavonoids might improve the transfection efficiency and cytotoxicity of lipoplexes via oxidative stress modulation. To test this hypothesis and obtain diverse information, we investigated the effects of eight flavonoids (Figure S1) which possess moderate log P values (0.75 ≤ log P ≤ 3.32, Table S1) on lipoplex (Lipofectamine 3000/plasmid DNA complexes)-mediated gene transfection in 2D and 3D cultures of murine colon carcinoma (Colon26) and human hepatocellular carcinoma (HepG2) cells. Flow cytometry and confocal microscopy were conducted to explore the relationship between oxidative stress, autophagy, and gene expression.

## 2. Materials and Methods

### 2.1. Materials

Epigallocatechin, myricetin, fisetin, quercetin, kaempferol, galangin, naringenin, and epigallocatechin gallate were purchased from the Tokyo Chemical Industry Co. (Tokyo, Japan). Lipofectamine 3000, CellROX Orange, and CellROX Deep Red were purchased from Thermo Fisher Scientific (Waltham, MA, USA), and the autophagy detection reagent, DALGreen, was purchased from Dojindo Laboratories (Kumamoto, Japan).

### 2.2. Preparation of Plasmid DNA

pcDNA3/GL encoding secretable *Gaussia* luciferase, ptdTomato-C1 encoding the red fluorescent protein tdTomato, and pTagBFP-N encoding the blue fluorescent protein TagBFP were purchased from Lux Biotechnology Ltd. (Edinburgh, UK), Clontech (Takara Bio Inc., Shiga, Japan), and Evrogen Joint Stock Company (Moscow, Russia), respectively. Amplification of plasmid DNA was performed using the *Escherichia coli* strain, DH5α. Plasmid DNA was purified using the EndoFree Plasmid Giga Kit (QIAGEN GmbH, Hilden, Germany). Cy5-labeled plasmid DNA was prepared using the Label IT Nucleic Acid Labeling Kit (Mirus Co., Madison, WI, USA).

### 2.3. Cell Culture

Colon26 and HepG2 cells were obtained from RIKEN (Tokyo, Japan). Colon26 cells were grown under standard conditions in Roswell Park Memorial Institute 1640 medium (Gibco; Thermo Fisher Scientific) supplemented with 10% fetal bovine serum (FBS), penicillin G (100 units/mL), and streptomycin (100 µg/mL) in a humidified atmosphere with 5% CO_2_ at 37 °C. HepG2 cells were grown under standard conditions in Dulbecco’s modified Eagle’s medium (Gibco) supplemented with 10% FBS, penicillin G (100 units/mL), and streptomycin (100 µg/mL) in a humidified atmosphere with 5% CO_2_ at 37 °C.

### 2.4. Preparation of Various Concentrations of Flavonoids

For epigallocatechin, treatment with less than 100 µM epigallocatechin could improve the apoptosis of PC12 cells, which were induced by 6-hydroxydopamine exposure [15]; incubation with 100 µM epigallocatechin also indicated decreased Jurkat cell viability [16]. For myricetin, treatments with over 25 µM myricetin induced the cytotoxicity of SNU-80 HATC cells [17]; it was also suggested that 30 µM myricetin showed a cytoprotective effect against hydrogen peroxide treatment through regulation of PI3K/Akt and MAPK signaling pathways [18]. For fisetin, 5–60 µM fisetin significantly increased the PC12 cell viability under cobalt chloride-induced hypoxic conditions [19]; it has also been reported that the flow cytometry of HepG2 cells treated with fisetin from 25 to 100 µM showed that fisetin decreased autophagic flux formation in a dose-dependent manner [20]. For quercetin, pre-incubation with 50 µM quercetin improved the mitochondrial function and retinal pigment epithelial cell viability after hydrogen peroxide treatment [21]; in addition, cellular autophagic activity induced by quercetin also showed a dose-dependent manner [22]. For kaempferol, a cytotoxic activity test was performed in RIN-5F cells, there was no cytotoxic effect till treated with 75 µM kaempferol [23]; on the other hand, 50 µM kaempferol inhibited autophagy-related proteins such as beclin-1 and p62/SQSTM1 [24]. For galangin, 25 and 50 µM galangin reduced the cellular ROS formation induced by 25 mM D–glucose [25]; in other research, treating HepG2 cells with 130 µM and 370 µM galangin could induce autophagy and cell apoptosis, respectively [26]. For naringenin, 25–100 µM naringenin efficiently scavenged paraquat-induced excessive ROS in BEAS-2B cells [27]; however, 100 µM naringenin caused nuclear changes with apoptosis in A432 cells [28]. For epigallocatechin gallate, co-treatment with hydrogen peroxide and 10 µM epigallocatechin gallate significantly increased IVD cell survival ratio via activation of the PI3K/Akt pathway [29]; besides, pretreatment with 10–50 µM epigallocatechin gallate effectively suppressed the reduction of retinal pigment epithelial cell viability [30].

According to the above references and results of our preliminary experiments, we finally decided on flavonoids concentrations as epigallocatechin (1.56–50 µM), myricetin (0.31–10 µM), fisetin (1.56–50 µM), quercetin (6.25–100 µM), kaempferol (3.13–100 µM), galangin (6.25–100 µM), naringenin (5–80 µM), and epigallocatechin gallate (6.25–100 µM). We weighed a certain weight of flavonoids, then dissolved the flavonoids with water (epigallocatechin, 1 mg/mL), or dimethyl sulfoxide (epigallocatechin gallate, 8 mg/mL; others, 5 mg/mL) to prepare the stock solutions, and diluted the stock solutions with OptiMEM (Gibco) to the appropriate concentrations.

### 2.5. Measurement of Gene Expression and Cell Viability

For 2D cultures, the cells were seeded into a 96-well plate at a density of 1 × 10^4^ cells/well and cultured for 24 h. The medium was replaced with the transfection medium, OptiMEM (Gibco) containing flavonoids and lipoplexes (200 ng/well pcDNA3/GL in Lipofectamine 3000). After 4 h, the cells were washed with phosphate-buffered saline (PBS, pH 7.4) and incubated with 10% FBS/medium for another 4 h at 37 °C.

For 3D cultures, the cells were seeded onto a 3D cell culture scaffold Cellbed (Japan Vilene Company, Ltd., Tokyo, Japan) in a 96-well plate format at a density of 5 × 10^4^ cells/well and cultured for 4 h, 3 d, and 7 d. Transfection was performed according to the same protocol used for the 2D cultures.

Transfection efficiency in cells was assessed by mixing 2 µL medium with 20 µL substrate (*Renilla* Luciferase Assay System containing *Gaussia* luciferase substrate coelenterazine; Promega, Madison, WI, USA), and bioluminescence levels were measured using a luminometer (Lumat LB 9507; Berthold Technologies, Bad Wildbad, Germany). Luciferase activity is indicated as relative light units (RLU)/mL of medium. After collecting the medium, each well was washed with PBS. Next, 100 µL of medium containing 10 µL of Cell Counting kit-8 (CCK-8; Dojindo, Kumamoto, Japan) was added. The cells were then incubated for 1 h. Absorbance was measured at 450 nm using a microplate photometer (Multiskan FC; Thermo Fisher Scientific). A medium without cells served as a negative control. For 3D cultures, the reactants of CCK-8 were transferred to another 96-well plate to avoid intrinsic absorbance of the scaffold.

### 2.6. Flow Cytometry

Cells were seeded into a 24-well plate at a density of 8 × 10^4^ cells/well, pre-incubated with the medium for 24 h, and washed thrice with PBS. The medium was then replaced with OptiMEM containing lipoplexes and ptdTomato-C1, with or without quercetin. After 4 h of incubation, the cells were washed thrice with PBS, incubated with CellROX Deep Red for 30 min, and washed again with PBS. Then, a fresh medium containing 10% FBS was added to the cells and incubated for an additional 12 h. After washing the cells twice with PBS containing 5% FBS, the cells were harvested by adding Trypsin-EDTA (0.5 mL) and incubating for 5 min. The cell suspension was centrifuged at 800× *g* for 5 min at 4 °C. The cells were washed twice with PBS containing 5% FBS, resuspended in the same solution, and analyzed by flow cytometry (LSRFortessa X-20; BD Biosciences, CA, USA).

### 2.7. Confocal Microscopy

HepG2 cells were seeded in 35 mm glass-based dishes at a cell density of 6 × 10^4^ cells/cm^2^ and pre-incubated for 24 h. Cells were washed thrice with PBS and pre-incubated with DALGreen for 30 min, according to the manufacturer’s protocol. After that, the cells were washed thrice with PBS and incubated with OptiMEM containing lipoplexes of pTagBFP-N/Cy5-labeled pTagBFP-N (0.5 µg each), with or without 25 µM quercetin, for 4 h. After washing the cells thrice with PBS, CellROX Orange was added to each dish. After 30 min of incubation, cells were washed thrice again and incubated with a fresh medium containing 10% FBS for a further 12 h. The samples were observed under a confocal laser scanning microscope (LSM710 with 405, 488, 543, and 633 nm laser lines; Carl Zeiss Microscopy GmbH, Jena, Germany).

### 2.8. Statistical Analysis

Statistical comparisons were performed using a one-way analysis of variance, followed by Dunnett’s post-hoc test for multiple comparisons with a control group. Statistical significance was set at *p* < 0.05.

## 3. Results and Discussion

### 3.1. Transfection Efficiency and Cell Viability with Flavonoids in 2D Colon26 Cells

**Figure 1:**
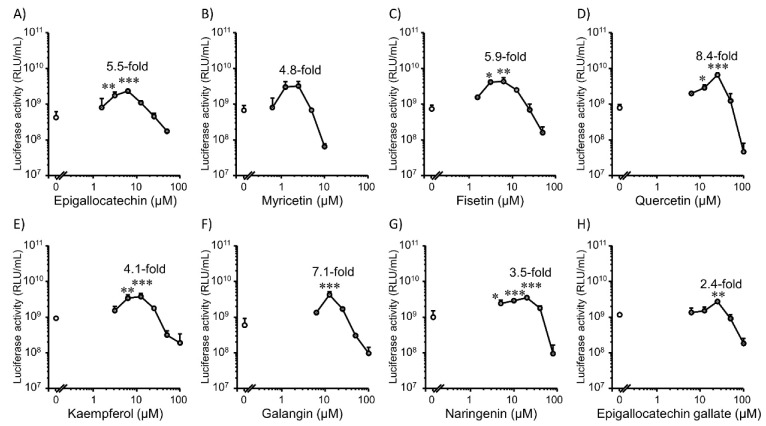
Effect of co-incubation with flavonoids on transfection efficiency mediated by Lipofectamine 3000/plasmid DNA (pcDNA3/GL 0.2 µg/well) complexes in 2D Colon26 cells. (**A**) Epigallocatechin (0–50 µM). (**B**) Myricetin (0–10 µM). (**C**) Fisetin (0–50 µM). (**D**) Quercetin (0–100 µM). (**E**) Kaempferol (0–100 µM). (**F**) Galangin (0–100 µM). (**G**) Naringenin (0–80 µM). (**H**) Epigallocatechin gallate (0–100 µM). Each dot represents the mean + standard deviation (SD; *n* = 3). * *p* < 0.05, ** *p* < 0.01, *** *p* < 0.001 compared with the control (0 µM flavonoids).

**Figure 2:**
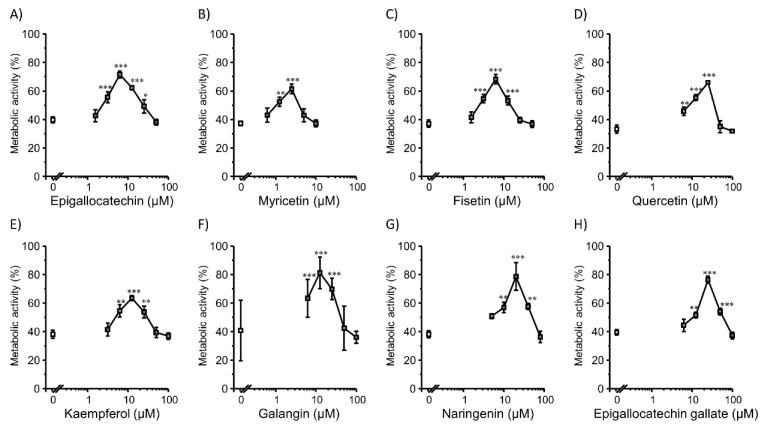
Metabolic activity of cells co-incubated with flavonoids and Lipofectamine 3000/plasmid DNA (pcDNA3/GL 0.2 µg/well) complexes in 2D Colon26 cells. (**A**) Epigallocatechin (0–50 µM). (**B**) Myricetin (0–10 µM). (**C**) Fisetin (0–50 µM). (**D**) Quercetin (0–100 µM). (**E**) Kaempferol (0–100 µM). (**F**) Galangin (0–100 µM). (**G**) Naringenin (0–80 µM). (**H**) Epigallocatechin gallate (0–100 µM). Each dot represents the mean ± SD (*n* = 3). * *p* < 0.05, ** *p* < 0.01, *** *p* < 0.001 compared with the control (0 µM flavonoids).

We tested the effect of co-incubation of flavonoids on the lipofection efficiency in 2D Colon26 cells (Figure 1). All tested flavonoids enhanced gene expression. The hydroxyl radical-scavenging ability is attributed to the structural elements of ring B in flavonoids [31], which may be important for enhancing gene expression. For all flavonoids, the transfection efficiency increased and then decreased with increasing flavonoid concentrations. In contrast, the CCK-8 assay showed a concentration-dependent increase in Colon26 metabolic activity (cell viability) when flavonoids were incubated at moderate concentrations (Figure 2). Decreased transfection efficiency and cell viability at high concentrations of flavonoids might be caused by the interruption of cellular redox homeostasis, which was induced by an extreme reduction in ROS. Also, the prooxidant property of a high concentration of flavonoids [32,33] might be a possible reason for decreased transfection efficiency and cell viability. Cationic lipid-based transfection also induces toxic effects in cells, which may lead to decreased transfection efficiency. Nicotinamide adenine dinucleotide phosphate (NADPH) oxidases [34,35,36] and lipoxygenase [37,38,39] are the most widely considered sources of ROS. NADPH oxidase activation is an initiating factor for mitochondrial damage, which ultimately leads to the death of HepG2 cells [40]. Lipoxygenase catalyzes the production of leukotrienes, which are sources of inflammation [41]. Quercetin (25 µM) showed the highest promotion effect for 8.4-folds compared to the control group (Figure 1D).

### 3.2. Transfection Efficiency and Cell Viability with Flavonoids in 2D HepG2 Cells

**Figure 3:**
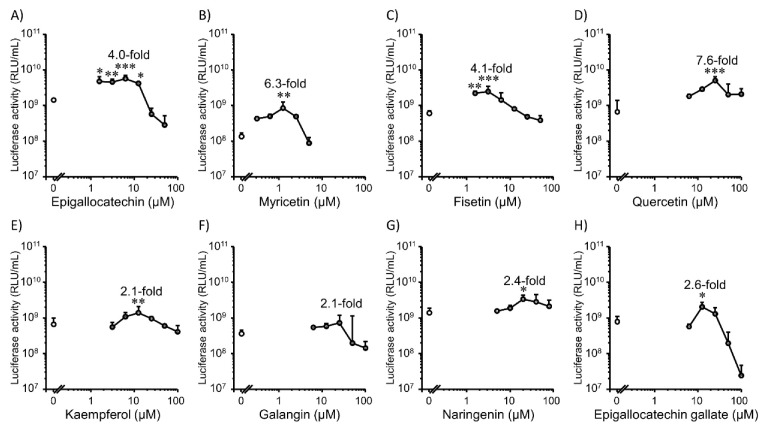
Effect of co-incubation with flavonoids on transfection efficiency mediated by Lipofectamine 3000/plasmid DNA (pcDNA3/GL 0.2 µg/well) complexes in 2D HepG2 cells. (**A**) Epigallocatechin (0–50 µM). (**B**) Myricetin (0–5 µM). (**C**) Fisetin (0–50 µM). (**D**) Quercetin (0–100 µM). (**E**) Kaempferol (0–100 µM). (**F**) Galangin (0–100 µM). (**G**) Naringenin (0–80 µM). (**H**) Epigallocatechin gallate (0–100 µM). Each dot represents the mean + SD (*n* = 3). * *p* < 0.05, ** *p* < 0.01, *** *p* < 0.001 compared with the control (0 µM flavonoids).

**Figure 4:**
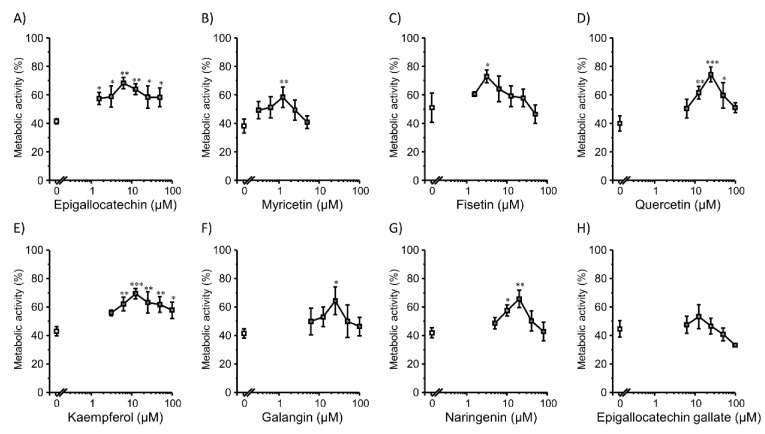
Metabolic activity of cells co-incubated with flavonoids and Lipofectamine^®^ 3000/plasmid DNA (pcDNA3/GL 0.2 µg/well) complexes in 2D HepG2 cells. (**A**) Epigallocatechin (0–50 µM). (**B**) Myricetin (0–5 µM). (**C**) Fisetin (0–50 µM). (**D**) Quercetin (0–100 µM). (**E**) Kaempferol (0–100 µM). (**F**) Galangin (0–100 µM). (**G**) Naringenin (0–80 µM). (**H**) Epigallocatechin gallate (0–100 µM). Each dot represents the mean ± SD (*n* = 3). * *p* < 0.05, ** *p* < 0.01, *** *p* < 0.001 compared with the control (0 µM flavonoids).

Next, we tested the effect of co-incubation with flavonoids on lipofection efficiency in 2D HepG2 cells (Figure 3). A similar trend was observed in Colon26 cells. Eight types of flavonoids increased the gene expression levels in 2D HepG2 cells. Moderate concentrations of flavonoids showed positive effects. Quercetin (25 µM) showed the highest promotion effect of 7.6-folds compared to the control group (Figure 3D). In addition, for myricetin (Figure 3B), galangin (Figure 3F), and epigallocatechin gallate (Figure 3H), the concentrations with the highest gene expression promotion effect were different compared to those for Colon26 cells (Figure 1B,F,H), indicating that the gene expression promotion effect of flavonoids may be cell type-dependent. Moderate concentrations of flavonoids also increased the viability of 2D HepG2 cells (Figure 4). 

We summarized the gene expression promotion effect of flavonoids in descending order (Table S2). Log P values of these flavonoids were all moderate. Not only log P values but also molecular weight is an important factor for uptake. Among tested flavonoids, epigallocatechin gallate has a larger molecular weight than other flavonoids. Therefore, uptake of epigallocatechin gallate might be inferior to those of others. Except for epigallocatechin gallate, the number of hydroxy groups in the B ring of highly potent flavonoids was 2 (catechol) or 3 (pyrogallol). Pannala et al. reported structure-activity relationships of flavonoids for antioxidant activity [42]. Their results showed B ring chemistry was critical for radical scavenging activity. Flavonoids with catechol showed rapid and prolonged radical scavenging activity compared with flavonoids with monophenol. In contrast, flavonoids with monophenol showed short radical scavenging activity. In addition, radical scavenging activity of flavonoids with unsubstituted B ring was not detected within the tested timescale (3 s). Thus, the radical scavenging activity of flavonoids may explain the different gene promotion effects obtained in this study. Because of the highest transfection efficiency in both 2D Colon26 and HepG2 cells, quercetin was chosen for further studies.

### 3.3. Transfection Efficiency and Cell Viability with Quercetin in 3D Colon26 Cells

**Figure 5:**
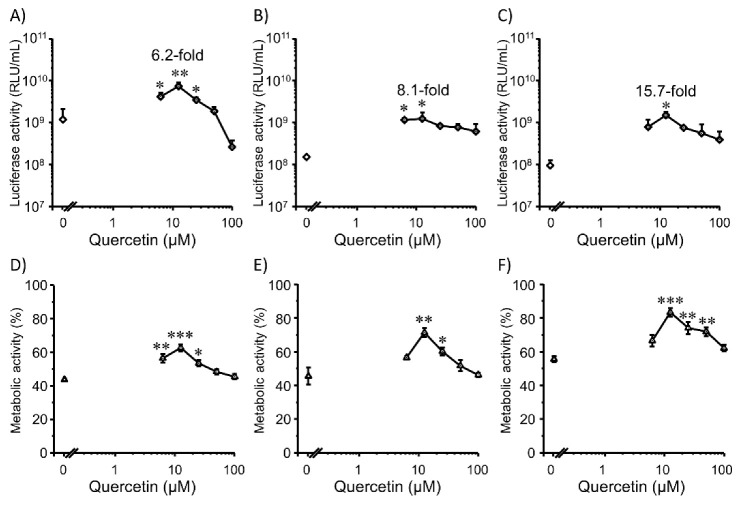
Effects of co-incubation with quercetin (0–100 µM) at three different transfection timings after seeding cells for 4 h (**A**,**D**), 3 d (**B**,**E**), and 7 d (**C**,**F**) on transfection efficiency (**A**–**C**) and metabolic activity (**D**–**F**) mediated by Lipofectamine 3000/plasmid DNA (pcDNA3/GL 0.2 µg/well) complexes in 3D Colon26 cells. Each dot represents the mean ± SD (*n* = 3). * *p* < 0.05, ** *p* < 0.01, *** *p* < 0.001 compared with the control (0 µM quercetin).

Although 2D cell cultures are still used in most studies, 3D cell culture has a broader range of applications as an analysis technology due to features that are closer to the complex in vivo conditions [43], proper cell-cell and cell-extracellular environment interactions [44], high metabolic enzyme expression [45], and possible long-term culture [46]. Therefore, we also investigated the transfection efficiency in 3D cultures of Colon26 cells. Transfection efficiency is strongly dependent on the cell cycle stage at the time of transfection [47], and gene expression responses may vary depending on the growth state and number of cells. Thus, transfection was also conducted at three different seeding times (4 h, 3d, and 7 d) (Figure 5A–C). The 3D cultures that we used here were of the scaffold type to perform a long-term culture study. From the results, 12.5 µM quercetin increased the gene expression level with increasing cell viability at all cell-seeding timings (Figure 5D–F). High concentrations of quercetin also decreased the transfection efficiency, similar to 2D cultures. The optimal concentration of quercetin was different in the 2D and 3D cultures. This can be attributed to the cell numbers as the cell numbers in 3D culture experiments were higher than those in 2D cultures. Therefore, the relative dose of lipoplexes per single cell in 3D cultures was lower than that in 2D cultures. Consequently, the oxidative stress induced by cationic lipoplexes in each cell of the 3D culture may be lower than that in 2D cultures. Thus, the concentration of quercetin, which is required to decrease ROS levels, may be lower in 3D cultures than in 2D cultures.

Three different time points were investigated after cell seeding. The results revealed different gene expression levels. Four hours after seeding, the cells showed the highest gene expression levels compared to the other two timings (Figure 5A). This may be attributed to the number and density of the cells, as follows: the number and density at 4 h after seeding cells should be lower than those at 3 d and 7 d (Figure 5B,C). At 4 h, the lipoplexes penetrated deep regions of the cell microenvironment due to low cell density. In addition, cell division can actively occur for 4 h, after which the plasmid DNA can enter the nucleus during mitosis. As expected, cell viability increased during the late stages of cell seeding (Figure 5E,F). Quercetin treatment increased the cell viability (Figure 5D–F) at moderate concentrations. Overall, the improved transfection efficiency may be due to improved cell activity.

### 3.4. Transfection Efficiency and Cell Viability with Quercetin in 3D HepG2 Cells

**Figure 6:**
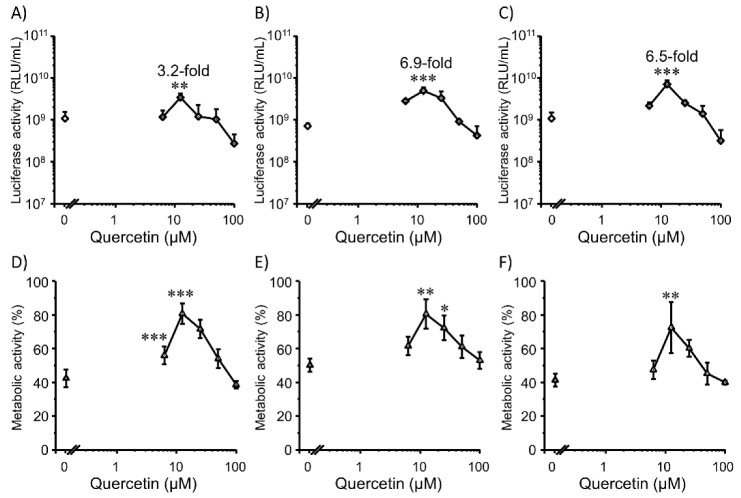
Effects of co-incubation with quercetin (0–100 µM) at three different transfection timings after seeding cells for 4 h (**A**,**D**), 3 d (**B**,**E**), and 7 d (**C**,**F**) on transfection efficiency (**A**–**C**) and metabolic activity (**D**–**F**) mediated by Lipofectamine 3000/plasmid DNA (pcDNA3/GL 0.2 µg/well) complexes in 3D HepG2 cells. Each dot represents the mean ± SD (*n* = 3). * *p* < 0.05, ** *p* < 0.01, *** *p* < 0.001 compared with the control (0 µM quercetin).

In 3D cultures of HepG2 cells, a trend similar to that in Colon26 cells was observed (Figure 6). Again, 12.5 µM quercetin showed the highest gene expression promotion effect at all three timings with improved cell viability. However, gene expression did not decrease during late cell seeding (3 and 7 d) (Figure 6B,C), which was different from the results in 3D Colon26 cells. Similar to the previous results, cell viability increased after treatment with quercetin (Figure 6D–F). Again, there was a different trend in HepG2 cells than in Colon26 cells in terms of cell viability. Cell viability in late cell seeding (Figure 6F) was not increased compared to that at 4 h (Figure 6D). The reason for the different trends between Colon26 and HepG2 cells in terms of transfection efficiency and cell viability is unclear, but cell death and proliferation at late phases may be more active in 3D HepG2 cells than in 3D Colon26 cells; consequently, nuclear pore disruption due to cell division guaranteed the nuclear entry of plasmid DNA in late cell seeding timings. In general, to choose the timing of cell seeding, we should consider the number of cells, cell density, and stage of the cell cycle for specific experimental needs. Flow cytometry was performed to explore the relationship between oxidative stress and transfection efficiency. 

### 3.5. Relationship between ROS Levels and Gene Expression of Lipoplexes

**Figure 7:**
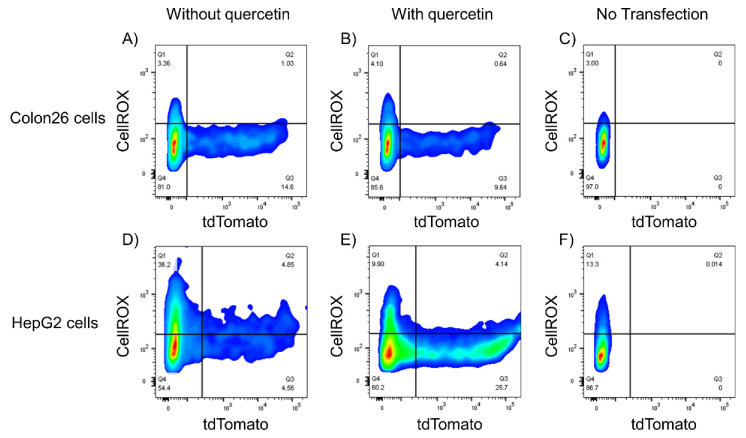
Relationship between gene expression and cellular reactive oxygen species (ROS) levels analyzed by flow cytometry. ptdTomato-C1 (1 µg/well) was complexed with Lipofectamine 3000 without (**A**,**D**) and with quercetin (25 µM) (**B**,**E**) transfected with lipoplexes, and cells without lipoplexes to detect basal ROS levels (**C**,**F**) in 2D Colon26 (**A**–**C**) and HepG2 (**D**–**F**) cells, respectively.

Using a flow cytometer, the relationship between ROS levels and gene expression was analyzed in 2D Colon26 and HepG2 cells (Figure 7). There were no differences between gene expression and ROS levels in the control and quercetin groups in Colon26 cells (Figure 7A,B). In contrast, in HepG2 cells, treatment with 25 µM quercetin reduced the population of gene expression-negative cells with high ROS levels and increased the number of gene expression-positive cells with low ROS levels (Figure 7D,E). We hypothesized possible reasons for the different results in the two cell lines as follows. Long-term incubation with quercetin may affect its stability, which leads to insufficient gene expression in Colon26 cells. However, this may not be the case in HepG2 cells; even after further incubation for 12 h, oxidative stress and gene expression levels still showed a good negative correlation. Therefore, not only the incubation time but also the cell type may affect the stability of quercetin. As reported in the literature, after different incubation times of quercetin in breast cancer (MCF-7) and colorectal carcinoma (HCT116) cells, a quantitative analysis of quercetin and quercetin metabolites in cell lysates revealed that both quercetin and quercetin metabolites showed different concentrations in different cell lines [48]. Thus, differences in cell type-dependent metabolism of quercetin may affect the promotion of transfection efficiency. In addition, the ROS level of gene expression-negative HepG2 cells was higher than that in Colon26 cells. HepG2 cells may be more susceptible to lipoplex-mediated ROS generation, and quercetin could work effectively in HepG2 cells.

Here, we selected HepG2 cells for observation by confocal microscopy in the next step.

### 3.6. Relationships among ROS, Autophagy, Cellular Uptake, and Transfection Efficiency

**Figure 8:**
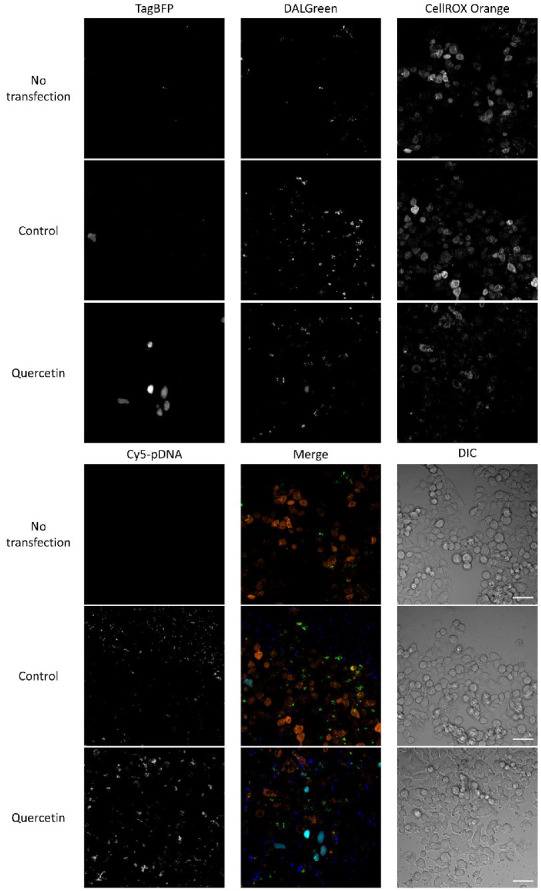
Relationships among transfection efficiency, autophagy, ROS, and cellular uptake in HepG2 cells observed by confocal microscopy. pTagBFP-N/Cy5-labeled pTagBFP-N (0.5 µg each/well) were complexed with Lipofectamine 3000. Concentration of quercetin was 25 µM. Cyan: gene expression (blue fluorescent protein TagBFP). Green: autophagy (DALGreen), Orange: ROS (CellROX Orange). Blue: plasmid DNA (Cy5-labeled pTagBFP-N). Acquisition conditions: TagBFP, laser, 405 nm (output, 0.2%; emission, 441–465 nm; master gain, 570); DALGreen, laser, 405 nm (output, 0.2%; emission, 518–537 nm; master gain, 600); CellROX Orange, laser, 543 nm (output, 50%; emission, 553–621 nm; master gain, 550); Cy5-labeled pTagBFP-N, laser, 633 nm (output, 38%; emission, 638–698 nm; master gain, 530). Objective lens was 25× LD LCI-plan-apochromat. Scale bar: 50 µm.

Figure 8 shows the relationships among oxidative stress, autophagy, cellular uptake, and gene expression in HepG2 cells. In the control experiment, transfection without quercetin showed relatively high ROS levels (CellROX Orange signals) compared with the no-transfection group, supporting that cationic lipoplexes can induce the production of ROS during transfection. In the quercetin group, gene expression-positive cells were accompanied by low ROS levels. This indicates that quercetin enhances exogenous gene expression via ROS regulation. On the other hand, cells with high autophagy levels (DALGreen signals) did not merge gene expression-positive cells in either the control or quercetin groups. This suggests that autophagy may be an inhibitory factor in transfection. In the control group, blue spots (plasmid DNA) were observed predominantly in the extracellular space, whereas the cellular association of plasmid DNA seemed to be low. The observation was performed 12 h after transfection. During this period, the majority of plasmid DNA in the cells may have been degraded. In the quercetin group, the cellular association plasmid DNA was more abundant than that in the control group. Meanwhile, green signals (autophagy) in the control group were many in number and strength, while those in the quercetin group were slightly fewer and weaker than those in the control group. Thus, autophagy may be more active in the control group than in the quercetin group; subsequently, plasmid DNA may be degraded more actively in the control group.

In this study, the correlation between ROS levels and gene expression was consistent with a previous study using edaravone [4]. There might be a continuous dynamic process involving ROS, inflammation, autophagy, and gene expression. High ROS production via lipofection may kill cells directly. Damage-associated molecular patterns (DAMPs), such as high-mobility group box-1, should be released. DAMPs can cause inflammation [49]. The plasmid DNA used in this study contained CpG motifs that are recognized by Toll-like receptor 9 [50]. ROS are associated with TLR activation [51], inducing inflammation. Cytosolic plasmid DNA may play the role of pathogen-associated molecular patterns, which can be recognized by the NLR family pyrin domain containing 3 (NLRP3) inflammasome, a component of the innate immune system [52]. Such an inflammatory spiral would further damage the cells. Quercetin may suppress these steps of cell death and inflammation and subsequently, stop the spiral.

Bacterial plasmid DNA can be recognized as foreign material by mammalian cells [53]. Activation of autophagy and the generation of tubulovesicular autophagosomes may lead to inefficient gene transfer, which is based on lipoplex- and polyplex-type non-viral vectors [54]. Therefore, during lipofection, lipoplexes may be degraded by activated autophagy, and autophagy may be considered an inhibitor of transfection. Indeed, degraded plasmid DNA should not be used for gene expression. However, if autophagy does not occur, cytosolic lipoplexes containing plasmid DNA can induce inflammasome activation. In contrast, autophagy can negatively regulate NLRP3 inflammasome activation by removing inflammasome activators [55]. It has been reported that 100 µM quercetin inhibits the NLRP3 inflammasome activation [56]. Therefore, a certain level of autophagy may be beneficial for transfection. Additionally, the main DNA sensors, cyclic guanosine monophosphate/adenosine monophosphate synthase (cGAS) and stimulator of IFN genes (STING), are essential pathways for immune reactions and type I interferon production [57]. STING is a regulator of cellular ROS homeostasis [58]. In addition, autophagy induced by the cGAS/STING pathway plays an important role in maintaining intracellular environmental homeostasis [59]. Therefore, the cGAS–STING pathway may also be involved in lipofection. Here, we propose a “moderate hypothesis” suggesting that moderate ROS, moderate inflammation and moderate autophagy levels can increase transfection efficiency. The nuclear import of plasmid DNA can occur during mitosis. However, nuclear import via nuclear localization signals through nuclear pores is still an active mechanism [60]. Moderate ROS levels and inflammation-mediated nuclear factor-κB activation may be involved in nuclear import, similar to a previous report [4]. Moderate autophagy may inhibit inflammasome activation, thereby improving cellular activity and enhancing gene expression. The fact that only a moderate concentration of quercetin exhibited improved transfection efficiency is consistent with this hypothesis.

We only selected a single time point for confocal observation and hypothesized that autophagy during transfection also involves the cell cycle because information on the whole transfection period was missing. As previously mentioned, the nuclear import of plasmid DNA can occur during mitosis. Autophagic flux is highly active during early mitosis [61,62]. Meanwhile, mitotic activity increased the transfection efficiency of lipoplexes, indicating that transfection close to the M phase may be promoted by nuclear membrane rupture [47]. Since the relationship between autophagy, cell cycle, and gene expression is complex, we suggest conducting live-cell imaging in further studies to obtain more comprehensive information on cells during the whole transfection process.

## 4. Conclusions

This study demonstrated the potential of eight flavonoids to increase the transfection efficiency of both Colon26 and HepG2 cells. Quercetin showed the highest promotion effect among tested flavonoids. Optimal concentrations of quercetin were different in 2D (25 µM) and 3D (12.5 µM) cultures. The promotion effect was achieved through scavenging excessive ROS and modulating autophagy to moderate levels, which was induced by cationic lipoplexes. Moderate regulation of ROS and autophagy levels is important for gene delivery. Further studies are needed to obtain more comprehensive information on the entire transfection process.

## Data Availability

All data are contained in the article.

## Supplementary Materials

The following are available online at: https://www.mdpi.com/article/10.3390/pharmaceutics14061203/s1, Table S1: Partition coefficients of flavonoids; Table S2: Summary of gene expression promotion effect and characteristics of flavonoids; Figure S1: Chemical structures of flavonoids.
