# Supplementary material for: Flavonoids Enhance Lipofection Efficiency and Ameliorate Cytotoxicity in Colon26 and HepG2 Cells via Oxidative Stress Regulation
Source: Pharmaceutics. 2022 Jun 5;14(6):1203. doi: 10.3390/pharmaceutics14061203 (PMC9231055; doi:10.3390/pharmaceutics14061203)
Supplement: Supplementary file 1 [file pharmaceutics-14-01203-s001.zip › pharmaceutics-1750558-supplementary.pdf]

Supplementary Information

Flavonoids Enhance Lipofection Efficiency and Ameliorate Cytotoxicity in Colon26 and HepG2 Cells via Oxidative Stress Regulation

Die Hu [1], Shintaro Fumoto [1,*], Hirotaka Miyamoto, Masakazu Tanaka [1], Koyo Nishida [1]

[1] Graduate School of Biomedical Sciences, Nagasaki University, Nagasaki 852-8501, Japan
* Correspondence: sfumoto@nagasaki-u.ac.jp; Tel.: +81-95-819-8568

Table S1. Partition coefficients of flavonoids

Flavonoids	Log P
Epigallocatechin	0.75-1.49*
Myricetin	1.42#
Fisetin	1.81-2.03*
Quercetin	1.81-2.16*
Kaempferol	1.99-2.46*
Galangin	3.322#
Naringenin	2.47-2.84*
Epigallocatechin gallate	2.38-3.08*

*: The University of Alberta and The Metabolomics Innovation Centre; #: PubChem, National Center for Biotechnology Information, National Institutes of Health.

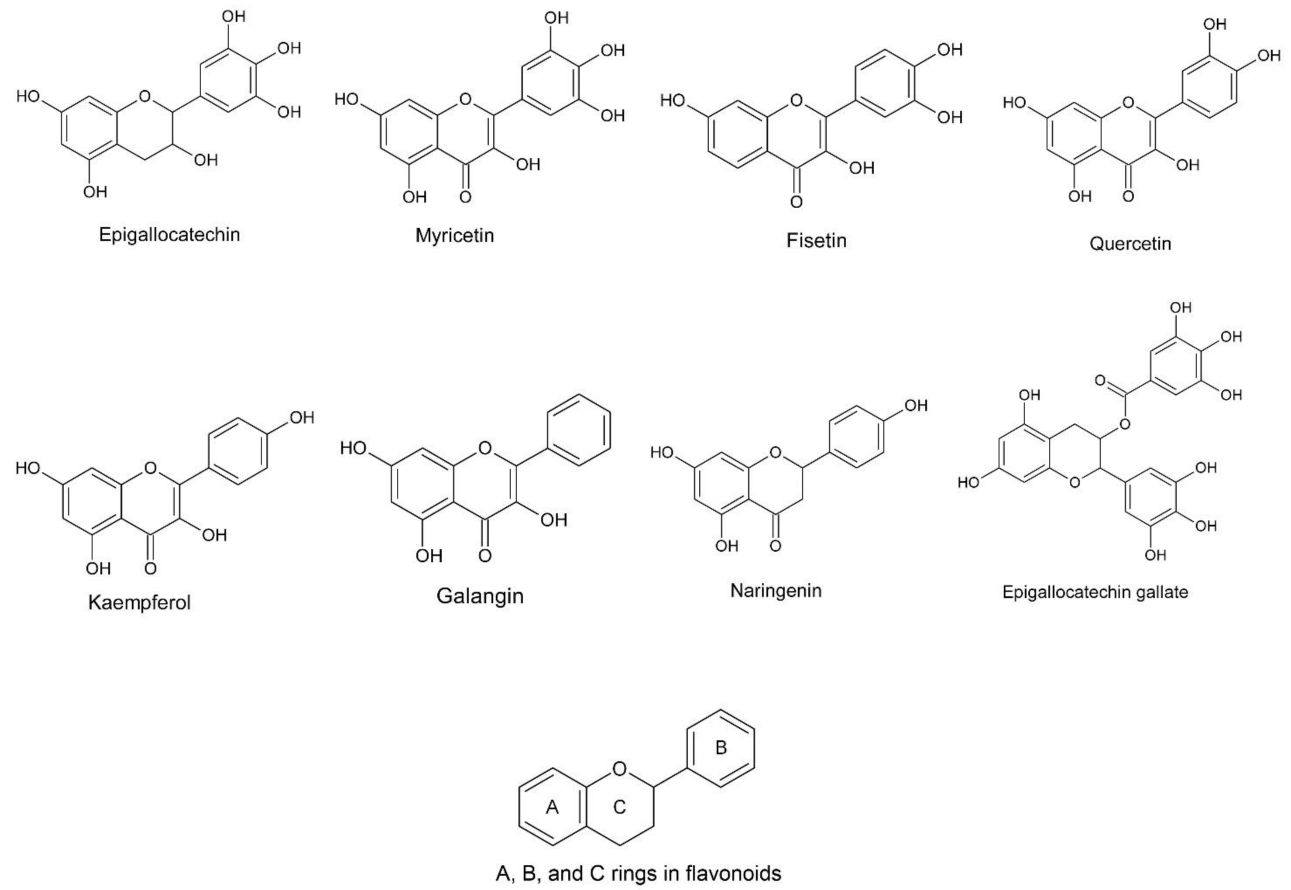

Figure S1. Chemical structures of flavonoids.

Table S2. Summary of gene expression promotion effect and characteristics of flavonoids

Flavonoids	Colon26 cells		HepG2 cells		Log P [*1]	Molecular weight	Number of OH			
	Fold increase	Conc. (μM)	Fold increase	Conc. (μM)			A ring [*2]	B ring [*2]	C ring [*2]	Total
Quercetin	8.4	25	7.6	25	1.81-2.16	302.236	2	2	1	5
Myricetin	4.8	2.5	6.3	1.25	1.42	318.235	2	3	1	6
Fisetin	5.9	6.25	4.1	3.13	1.81-2.03	286.236	1	2	1	4
Epigallocatechin	5.5	6.25	4.0	6.25	0.75-1.49	306.27	2	3	1	6
Galangin	7.1	12.5	2.1	25	3.322	270.24	2	0	1	3
Kaempferol	4.1	12.5	2.1	12.5	1.99-2.46	286.23	2	1	1	4
Naringenin	3.5	20	2.4	20	2.47-2.84	272.257	2	1	0	3
Epigallocatechin gallate	2.4	25	2.6	12.5	2.38-3.08	458.38	2	3	0	8

*1: Identical with Table S1. *2: Ring positions are indicated in Figure S1.
